# Feminization and Stress in the Veterinary Profession: A Systematic Diagnostic Approach and Associated Management

**DOI:** 10.3390/bs9110114

**Published:** 2019-11-14

**Authors:** Lisa Emmett, Jan Aden, Anastasiya Bunina, Armin Klaps, Birgit U. Stetina

**Affiliations:** 1 Psychological Outpatient Clinic, Sigmund Freud University Vienna, 1020 Vienna, Austriabirgit.u.stetina@sfu.ac.at (B.U.S.); 2 Psychological Faculty, Sigmund Freud University Vienna, 1020 Vienna, Austria; jan.aden@sfu.ac.at (J.A.); anastasiya.bunina@sfu.ac.at (A.B.)

**Keywords:** female veterinarians, stress, coping

## Abstract

Within the field of veterinary medicine the gender distribution has changed, since most graduates are now females. Studies show that female veterinarians represent a vulnerable group for stress and stress-related illnesses. The goal of the study was to identify typical profiles of stress management strategies and to clarify if vets are well-equipped to cope with occupational stressors. Within a cross-sectional design 78 female veterinarians from Austria and Germany were surveyed using a self-report test-battery assessing twenty different coping styles. Statistical analysis included one sample *t*-tests and Cohen’s d as a concurrent effect size measure. The results showed that female veterinarians are significantly more likely to use negative coping styles for their stress e.g., rumination (*t*(74) = 6.733, *p* = < 0.001, *d* = 0.726) or escapism (*t*(72) = 2.173, *p* = 0.033, *d* = 0.281) when compared to the norm population. Amongst other studies these findings contribute to a systematic diagnosis which is necessary for the development and implementation of standardized stress management interventions for the veterinary education and training e.g., courses for improving communication and stress management skills and regular supervision or intervision (exchange with professional peers). Due to existing stigmatization concerning mental health, low-barrier counseling services should be provided for veterinarians who already feel stressed.

## 1. Introduction

The gender distribution has fundamentally changed in the veterinary field. In Vienna for example, about 78% of students are female graduates [1]. These statistics are consistent to data of other countries like Canada and the USA where about 80% of graduates are female [2]. Although until now the gender distribution among practicing veterinarians was rather balanced (e.g., Germany: about 51% [3]; Austria: about 49% [4]) it can be expected to change in the next years. Moreover, in reference to gender differences and stress, studies show that females are more prone to somatic stress reactions [5]. Furthermore, they experience additional stressors in comparison to their male colleagues (e.g., salary gap). In this context it is highly relevant to consider that health professionals per se seem to be a vulnerable group for stress and stress related illnesses, as they are confronted with psychological stress on a daily basis [6], which in turn may be a serious health hazard [7]. In case of veterinarians it is obvious that these professionals act as an interface between handler and animals and therefore have to deal with both human and animal side. Within this triad veterinarians have to show high commitment (socially and emotionally) which is necessary to gain the trust of handlers, in regards to their professional expertise and associated recommended interventions. Consequently, a very important occupational aspect seems to be the communication with the handler. This, especially in cases of difficult clients, can cause a lot of stress [8]. Nevertheless, these essential competences are barely taught throughout their education and training, even though high communication skills may be related to less stress susceptibility, as well seem to be associated with higher professional success [9]. 

Among practicing veterinarians studies show that they have to cope with various stressors regarding communication (as stated before), death and dying, work conditions, business practices and individual factors. Professionals, for example, have to deal with: angry or emotional clients, violence, euthanasia, long working hours [10]. Also, personal stress related factors had been identified like reduction in psychological health (e.g., depression or anxiety), suffering from insomnia or sleeping difficulties, greater conflict in managing the demands of work and private life as well as reduced job satisfaction [11]. Stress, however, not only affects practicing veterinarians. There is also evidence that young, recent graduates show higher levels of stress, depression and anxiety [6]. But even veterinary students experience a lot of stress, as they have to learn about the diagnosis and treatment of a host of different animals, as well have to cope with stressful experiences like pet loss [12]. Summarizing it can be noted that throughout their entire career veterinarians experience a lot of stress. Studies also support the idea that female veterinarians suffer more from mental strain and stress than their male counterparts [6,13]. Again, also throughout their education gender-specific differences can be documented as for example experiencing pet loss also seems to be a more common distress for female students [12].

Being exposed to stress on a longer period of time obviously has serious effects on health. Therefore, stress can act as a risk factor for additional health problems like consumption of psychotropic substances or even suicidal tendencies. Some studies have already concluded that there is a significant correlation between work related stress and problematic alcohol consumption in men [14]. In the case of veterinarians Harling and colleagues found, that examples of women practicing high risk consumption of alcohol are more frequent than males [15]. Regarding suicidal tendencies Nett and colleagues [16] found that female veterinarians practicing in the US show increased suicidal ideation (19.1%) compared to female adults (7.1%). It can furthermore be argued that there may be a connection between the major method of suicide in the form of poisoning and the easy access veterinarians have to drugs [17]. Compared to the general population a clear distinction can be observed, as the major method seems to be hanging [18]. 

Especially in the field of stress management there is a wide range of effective psychological treatment available [19] in order to prevent for example subsequent health risks (e.g., substance abuse). But unfortunately, even within health professionals e.g., physicians, there are observable stigmas concerning mental health [20], which seem to be a barrier for seeking treatment. For example, a study on attitudes of doctors on becoming mentally ill showed that about 73% would choose to disclose a mental illness to their family rather than to a professional [21], which is consistent with the findings verifying veterinarians seeking support within their social environment rather than in the form of professional counseling [22]. These stigmas and associated barriers to health offers obviously have negative effects on prevention and secondary prevention in view of mental illnesses.

Summarizing, it can be said that female veterinarians (within their education and throughout their career) represent a vulnerable group for stress as well as stress related illnesses. The circumstance of a changing gender distribution, specifically more women and women who are or will be in their child bearing years, should also be considered [6]. As stated above, this enhanced level of stress perceived may have an effect on the health condition and could thus have negative effects on subsequent medical care. The aim of the study was to identify a profile of typical coping strategies in female veterinarians. In addition, it was explored if this professional group is well-equipped to cope with their professional conditions and associated stressors. Finally, it was explored as an example if the examined population is significantly more likely to use medication (usage of substances) as a means of stress management. 

## 2. Materials and Methods

### 2.1. Ethics of Data Collection

All subjects gave their informed consent for inclusion before they participated in the study. The study, which is part of an ongoing research project that started in 2016, was approved by the Sigmund Freud University ethical committee (SBHVGD9RAGUFC887564).

### 2.2. Participants

Inclusion criteria required participants to be female, German speaking and having already completed their veterinary university studies, as well as working as veterinarians. There were no restrictions regarding the veterinary specialty or number of years of professional experience. 

### 2.3. Instruments

To measure coping strategies a self-report test battery called Questionnaire on Coping Strategies (Stressverarbeitungsfragebogen, SVF-120) [23] was used. It consists of 120 items and measures twenty different coping strategies. This instrument has a good quality criteria. T-values between 40 and 60 can be considered as average. Values lower 40 demonstrate a low characteristic of the respective strategy. In contrast to values over 60 which speak for a high characteristic of the respective strategy. The measured coping strategies can be divided in two subscales called positive (POS) and negative (NEG) coping styles. Following strategies are assigned to the subscale POS as they help to reduce someone’s stress level: minimization, downplay, deflecting guilt, distraction, compensatory satisfaction, self-affirmation, relaxation, situation control as well as positive self-instruction. In contrast to strategies, which are part of the subscale NEG Strategies because they lead to a constant or even higher stress level for example: escapism, social encapsulation, rumination, resignation, self-pity, self-incrimination. Finally, following coping strategies are not assigned to the positive or negative subscale: avoidance, need for social support, aggression and medication.

### 2.4. Statistical Methods

All analyses were conducted using IBM SPSS Statistics Version 25 (SPSS Inc., Chicago, IL, USA) and the significance level was set at *p* ≤ 0.05. Sociodemographic data were reported in frequencies and percentage. Subsequent statistical analysis included one sample *t*-tests. Accordingly, every subscale of the SVF-120 was compared to the means of the norm population, Cohen’s d was used as a concurrent effect size measure.

## 3. Results

Using a cross-sectional design an online study was conducted to survey 78 female veterinarians. The population was aged from 24 to 64 years (M = 38.29, SD = 9.13). Concerning the marital status, the sample was mainly in a relationship (43.6%, N = 34) or married (30.8%, N = 24). The rest of the sample was divorced, single or living apart (25.6%, N = 20). In regard to veterinary specialty the most popular were small animals (about 71.8%, N = 56) The remaining 22.2% (N = 22) worked in following fields: big animals, surgery, laboratory diagnostics, pathology, research, teaching, official veterinarian, food control, animal disease control, animal welfare, industry, etc. Most of the respondents stated that they were employees (62.8%, N = 49). 34.6% (N = 27) were self-employed and only 2.6% (N = 2) said that they were part employed and/or self-employed. The average working hours per week were 43.91 (SD = 14.22).

Several groups of job stressors were identified, but only 70 women of the sample responded to the open-ended question concerning occupational stressors. The most relevant ones were: communication with animal owners/handlers (54.3%, N = 38), emergency services with 24/7 availability (10.0%, N = 7) and the well-known stressor euthanasia (11.4%, N = 8). The remaining 24.3% (N = 17) referred amongst other stressors to bookkeeping/office work and animal welfare. Subsequent statistical analyses included a comparison of every subscale of the SVF-120 to means of the norm population by calculating one sample *t*-tests and Cohen’s d as a concurrent effect size. The results showed the following coping profiles: Regarding negative coping strategies (lead to a constant or higher stress level) veterinarians had a significant tendency to use negative, maladaptive coping strategies when they felt stressed. Compared to the norm population these professionals tend more to escape (*t*(72) = 2.173, *p* = 0.033, *d* = 0.281), ruminate (*t*(74) = 6.733, *p* = < 0.001, *d* = 0.726) resignate (*t*(76) = 2.541, *p* = 0.013, *d* = 0.316), pity themselves (*t*(74) = 3.140, *p* = 0.002, *d* = 0.388) and incriminate themselves (*t*(76) = 2.374, *p* = 0.020, *d* = 0.301) when they are confronted with stressful situations (see Table 1). Therefore, they react to stress related situations in the following ways: not being able to distance themselves from stressful thoughts (*rumination*) and giving up with feelings of helplessness and hopelessness (*resignation*).

In addition, they are significantly less likely to apply positive, healthy coping strategies e.g., minimization (*t*(76) = −5.706, *** *p* = < 0.001, *d* = 0.671), downplay (*t*(70) = −3.778, *** *p* = < 0.001, *d* = 0.473), relaxation (*t*(75) = −2.683, ** *p* = 0.009, *d* = 0.313), reaction control (*t*(74) = −2.766, ** *p* = 0.007, *d* = 0.330), as well as show positive self-instruction (*t*(76) = −5.759, *** *p* = < 0.001, *d* = 0.578) (see Table 2). Accordingly, the examined sample is significantly less likely to use the following strategies: devaluate strength, duration or seriousness of a strain (*minimization*), attribute to themselves less stress than to others (*downplay*), being able to relax in general or single body parts (*relaxation*), bring and keep their own reaction under control (*reaction control*) as well as attribute themselves competence and control capability (*positive self-instruction*).

Finally, the examined sample is more likely to show aggression (*t*(74) = 3.365, *** *p* = < 0.001, *d* = 0.399) and tend to look for social support and help (*need for social support*) (*t*(74) = 5.199, *** *p* = < 0.001, *d* = 0.088) when they feel stressed. There can be no significant differences documented in view of the subscales avoidance *t*(73) = 0.522, *p* = 0.603) or medication *t*(74) = 0.510, *p* = 0.611). 

## 4. Discussion

### 4.1. Academic Contribution and Practical Implications

A large number of studies were able to demonstrate that a high percentage of veterinarians and especially female veterinarians experience work related stress [6]. The most common causes seem to be too heavy workload, insecurity of the work contract or being on call [13]. Moreover, communication with clients and associated expectations also seem to be relevant stressors [23].

The aim of the present study was to examine relevant stress management strategies within a sample of female veterinarians to find out if these professionals are well-equipped to cope with these occupational stressors they encounter on a daily basis. Accordingly, by using a self-report battery (SVF-120), a typical stress management profile including stress-reducing as well as stress-increasing strategies were determined. The results of the present study show that female veterinarians use a variety of negative stress management strategies, which can cause several health problems in the long term. The present findings of maladaptive coping mechanism in female veterinarians may also result from present working conditions and associated stressors. 

When thinking about effective stress management, spanning several professional groups, following relevant steps can be suggested: (a) stress diagnosis, (b) intervention choice and design, (c) implementation and (d) evaluation [14]. A host of relevant studies have been published concerning the first step *stress diagnosis* [6,22,24]. These findings refer to certain occupational stressors (environmental factors), as well as the present study to typical coping profiles in veterinarians (intraindividual factors). Very recent studies also focus on developing adequate instruments for measuring potential predictors of burnout and engagement of young veterinarian professionals based on the Job-Demands-Resources model [25]. This model distinguishes employee wellbeing in terms of job demands (e.g., work overload, work-home interference, shift work and role ambiguity) and job resources (e.g., autonomy, social support, career opportunities) [26]. Moreover, exhaustion, in-role performance (task oriented), work engagement, extra role performance (organization oriented) are included in the model [27]. The JD-R model has then been extended by the factor personal resources, which can be described as an aspect of the self that is linked to resiliency [28] (being able to manage crises and gain access to personal resources and using it for development). This standardized approach not only facilitates cross-national comparisons but contributes to derive possible interventions. 

This systematic diagnosis approach based on recent scientific findings is highly relevant to choosing from different intervention types, as well as planning adequate interventions [29]. Based on the systematic diagnosis, stress management interventions within the veterinary profession should start at the following relevant areas: (1) reduction of job demands, (2) enhancement of job resources and (3) improvement of personal resources [25]. Additionally, we propose that a larger focus should be placed on the level of secondary prevention: support possibilities for risk groups (4).

(1) *Reduction of job demands*: There is evidence, especially in the field of the veterinary profession, that long working hours and associated lack of off time represent significant stressors [29]. Accordingly regulated working hours and sufficient periods of recovery seem to be an essential first starting point for stress management.
(2) *Enhancement of job resources*: Secondly, it is highly recommended to establish the essential framework for intervision and supervision for the veterinary profession in order to give this professional group the opportunity for constant exchange with experienced colleagues and receiving feedback and support.
(3) *Improvement of personal resources*: Moreover, trainings for communication and stress skills should be considered during the university education, in the form of lectures concerning the following issues: work-life balance, stress management, development of procedures in view of communication with (difficult) clients. Very relevant work has been published concerning the need for educating university staff on the consequences of stress [12]. Especially regarding giving bad news (e.g., delivering news about unexpected pet loss or chronic or terminal illness) there is evidence that this difficult conversational situation gets easier with practice and that repeated exposure will help to develop strategies to cope with associated behavioral and emotional challenges [30]. As mentioned before, repeated exposure should be part of lectures within the veterinary training. Improving communication skills may not only reduce stress, but might also lead to positive effects on professional practice in form of optimizing internal communication (e.g., planning projects, communicating with colleagues) as well as external communication (with handlers) [9]. This latter aspect of work optimization not only applies to communication but to all areas of stress management.
(4) *Support possibilities for risk groups*: Planned stress management interventions should also offer opportunities for veterinarians, who already feel stressed and professional support in form of secondary prevention is indicated. Because of existing stigmatization within health professionals regarding mental health, it is important to provide preferably low-barrier counseling services. This would make it easier for veterinarians to get in contact with mental health care services. For this reason, beside help lines, bibliotherapy or self-help materials online, counseling would be an adequate opportunity. Advice-seeking individuals would have the chance to seek consultation anonymously and not be bound to specific times and locations [31].

Finally, all the above-mentioned stress management interventions need to be evaluated to test if these target the relevant points and lead to a reduction of perceived stress. For this purpose, standardized instruments (e.g., Questionnaire on Coping Strategies (Stressverarbeitungsfragebogen, SVF-120) [23]; Vet-DRQ [25]) requiring at least two measurement time points need to be used to verify if established trainings for communication and stress management skills in fact lead to a significant improvement. 

### 4.2. Limitations and Future Research Directions

Possible limitations of the study might be the sample size. Future studies should use standardized instruments for identifying typical coping profiles within a bigger sample. In addition, it could be argued that the way which veterinarians react in stressful situations may be linked to the category of stressor they are confronted with (e.g., handler communication or workload). These aspects should be considered in the context of future research. In conclusion, female vets use a lot of negative stress management strategies to cope with the stressors they encounter on a daily basis. Standardized procedures in regard to stress diagnostics, selection and implementation of stress management strategies and their evaluation could help veterinarians cope with their occupational stress. Therefore, the next step should be to develop standardized trainings that help female veterinary students and female veterinarians improving their stress management und communication skills in terms of mental health prevention as well as professional success (e.g., improvement of problem-solving skills, decision making and time management) [12].

## Figures and Tables

**Table 1:** Negative coping strategies of the sample compared to the norm population.

Coping Strategies	*t*	df	*p*-Value
escapism	2.173	73	0.033
social encapsulation	1.943	75	0.056
rumination	6.733	74	0.001
resignation	2.541	75	0.013
self-pity	3.140	74	0.002
self-incrimination	2.374	75	0.020

**Table 2:** Positive coping strategies of the sample compared to the norm population.

Coping Strategies	*t*	df	*p*-Value
minimization	−5.706	75	0.001
downplay	−3.778	70	0.001
deflecting guilt	−1.680	74	0.097
distraction	−1.047	72	0.298
compensatory satisfaction	1.452	74	0.151
self-affirmation	−1.218	74	0.227
relaxation	−2.683	74	0.009
situation control	−0.417	73	0.678
reaction control	−2.766	74	0.007
positive self-instruction	−5.759	75	0.001

## References

[1] Jahresbericht Veterinärmedizinische Universität. https://www.vetmeduni.ac.at/fileadmin/v/z/universitaet/vetmedunijahresbericht2017_de_screen.pdf.

[2] Lofstedt J. (2003). Gender and veterinary medicine. Can. Vet. J..

[3] Statistik der Bundestierärztekammer. https://www.bundestieraerztekammer.de/btk/statistik/.

[4] Österreichisches Institut für Wirtschaftsforschung Wirtschaftliche Grundlagen für Strategische Entscheidungen zur Zukunft der Veterinärmedizin in Österreich—Bericht. https://www.tieraerztekammer.at/fileadmin/daten/downloads/Bericht_WIFO_09_10_2012.pdf.

[5] Matud M.P. (2004). Gender differences in stress and coping styles. Personal. Individ. Differ..

[6] Shirangi A., Fritschi L., Holmann C.D.J., Morrison D. (2018). Mental health in female veterinarians: Effects of working hours and having children. Aust. Vet. J..

[7] Shapiro S.L., Astin J.A., Bishop S.R., Cordova M. (2005). Mindfulness-based stress reduction for health care professionals: Results from a randomized trial. Int. J. Stress Manag..

[8] Milani M. (2007). The Art of Private Veterinary Practice: The Art of Apology. Can. Vet. J..

[9] Kleen J.L., Rehage J. (2008). Kommunikationskompetenz in der tierärztlichen Praxis. Tierarztl. Prax. Ausg. G.

[10] (2002). Survey details stress factors that influence Australian vets. Aust. Vet. J..

[11] Meehan M.P., Bradley L. (2007). Identifying and evaluating job stress with the Australian small animal veterinary profession. Aust. Vet. Pract..

[12] Gelberg S., Gelberg H. (2005). Stress management interventions for veterinary students. J. Vet. Med Educ..

[13] Hatch P.H., Winefield H.R., Christie B.A., Lievaart J.J. (2011). Workplace stress, mental health and burnout of veterinarians in Australia. Aust. Vet. J..

[14] Bobak M., Pikhart H., Kubinova R., Malyutina S., Pajak A., Sebakova H., Marmot M. (2005). The association between psychosocial characteristics at work and problem drinking: A cross-sectional study of men in three Eastern European urban populations. Occup. Environ. Med..

[15] Harling M., Strehmel P., Schablon A., Nienhaus A. (2009). Psychosocial stress, demoralization and the consumption of tobacco, alcohol and medical drugs by veterinarians. J. Occup. Med. Toxicol..

[16] Nett R.J., Witte T.K., Holzbauer S.M., Elchos B.L., Campagnolo E.R., Musgrave K.J., Karter K.K., Kurkjian K.M., Vanicek C., O’Leary D.R. (2015). Prevalence of Risk Factors for Suicide Among Veterinarians—United States, 2014. MMWR Morb. Mortal. Wkly. Rep..

[17] Jones-Fairne H., Ferroni P., Silburn S., Lawrence D. (2008). Suicide in Australian veterinarians. Aust. Vet. J..

[18] Australian Bureau of Statistics Suicides: Recent Trends, Australia. http://www.abs.gov.au/ausstats/abs@.nsf/mf/3309.0.55.001.

[19] Kaluza G. (2008). Gelassen und Sicher im Stress: Das Stresskompetenz-Buch: Stress Erkennen, Verstehen, Bewältigen.

[20] Fink-Miller E.L., Nestler L.M. (2018). Suicide in physicans and veterinarians: Risk factors and theories. Curr. Opin. Psychol..

[21] Hassan T.M., Ahmed S.O., White A.C., Galbraith N. (2009). A postal survey of doctors’ attitudes to becoming mentally ill. Clin. Med..

[22] Gardner D.H., Hini D. (2006). Work-related stress in the veterinary profession in New Zealand. N. Z. Vet. J..

[23] Erdmann G., Janke W. (2008). Stressverarbeitungsfragebogen: SVF; Stress, Stressverarbeitung und Ihre Erfassung durch Ein mehrdimensionales Testsystem.

[24] Rejula K., Räsänen K., Hämäläinen M., Juntunen K., Lindbohm M.L., Taskinen H., Rinta-Jouppi M. (2003). Work environmental and occupational health of Finnish veterinarians. Am. J. Ind. Med..

[25] Mastenbroek N.J.J.M., Demerouti E., Van Beukelen P., Muijtjens A.M., Scherpbier A.J.J.A., Jaarsma A.D.C. (2013). Measuring potential predictors of burnout and engangement among young veterinary professionals; construction of a customized questionnaire (the Vet-DRQ). Vet. Rec..

[26] Bakker A.B., Demerouti E., Euwema M.C. (2005). Job Ressources Buffer the Impact of Job Demands on Burnout. J. Occup. Health Psychol..

[27] Bakker A.B., Demerouti E., Verbeke W. (2004). Using the job demands-ressources model to predict burnout and performance. Hum. Ressour. Manag..

[28] Hobfoll S.E., Johnson R.J., Ennis N., Jackson A.P. (2003). Resource loss, resource gain, and emotional outcomes among inner city woman. J. Personal. Soc. Psychol..

[29] Smith D.R., Leggat P.A., Speare R., Townley-Jones M. (2009). Examining the dimensions and correlates of workplace stress among Australian veterinarians. J. Occup. Med. Toxicol..

[30] Ptacek J.T., Leonard K., McKee T.L. (2004). “I’ve Got Some Bad News…”: Veterinarians’ Recollections of Communicating Bad News to Clients 1. J. Appl. Soc. Psychol..

[31] Knatz B., Kühne S., Hintenberger G. (2011). Die webbasierte Mailberatung. Handbuch der Online Beratung: Psychosoziale Beratung im Internet.

